# Brief mechanical pulses induce sustained intracellular L-lactate production in astrocytes

**DOI:** 10.3389/fncel.2026.1830831

**Published:** 2026-06-17

**Authors:** Kaja Belko Parkel, Danaja Kuhanec, Živa Tajda Gržina, Helena Haque Chowdhury, Robert Zorec, Marko Kreft

**Affiliations:** 1 Laboratory of Neuroendocrinology-Molecular Cell Physiology, Institute of Pathophysiology, Faculty of Medicine, University of Ljubljana, Ljubljana, Slovenia; 2 Celica Biomedical, Ljubljana, Slovenia; 3 Department of Biology, Biotechnical Faculty, University of Ljubljana, Ljubljana, Slovenia

**Keywords:** astrocytes, FRET, lactate, metabolism, TRPV4, mechano-metabolic coupling

## Abstract

Intracranial-pressure transients impose mechanical strain on perivascular astrocytes, but it is unclear whether brief mechanical events elicit metabolic responses. Here, we applied 1.5-s pressure-driven mechanical stimuli (5–1,000 hPa) to cultured primary rat cortical astrocytes using a patch-micropipette microinjector and monitored cytosolic lactate ([lactate]_i_) with a Förster resonance energy transfer Laconic nanosensor. Single pressure pulses (5–1,000 hPa) evoked a pressure-dependent increase in [lactate]_i_ that persisted for minutes after stimulation, reaching a steady-state increase of 0.3–4.1% relative to baseline over 200–300 s. Sequential pulses delivered to the same cell produced transient spikes followed by stepwise increases in the post-stimulus plateau, yielding an estimated activation threshold of ~4–5 hPa. Transient receptor-potential vanilloid-4 (TRPV4) immunoreactivity was detected in cultured astrocytes, and pharmacologic inhibition of TRPV4 with HC-067047 attenuated mechanically evoked lactate accumulation during mechanical stimulation by solution bolus addition. These findings demonstrate that astrocytes convert brief mechanical stimuli into prolonged cytosolic lactate increases, supporting a contribution of TRPV4-associated mechanosensitive signaling to astrocytic mechano-metabolic integration.

## 1 Introduction

Cells throughout the body, including those in the skin and brain, sense mechanical stress and adjust metabolism to support surrounding tissue (Xue et al., 2025). In the brain, energy-intensive information processing relies on tightly regulated oxygen and nutrient delivery, maintained by complex blood flow regulation (Attwell et al., 2010; Willie et al., 2014; Hosford and Gourine, 2019). Fluctuations in intracranial pressure during cerebral perfusion stress perivascular astrocytes, but it is unknown whether these homeostatic, energy-supporting cells respond to these fluctuations by altering metabolism (e.g., aerobic glycolysis and L-lactate production).

Decreases in systemic blood pressure or increases in intracranial pressure can restrict cerebral blood flow, causing reduced cerebral perfusion pressure, which may lead to cerebral hypoperfusion and subsequent inadequate delivery of essential nutrients and oxygen to brain tissue (Peterson et al., 2011). Emerging evidence from studies on mice (Choi et al., 2015; Schmidt et al., 2018; Haidey et al., 2021) and rats (Kim et al., 2015; Marina et al., 2020; Turovsky et al., 2020) suggests an intrinsic brain mechanism capable of detecting physiologic reductions in cerebral perfusion pressure. This mechanism triggers compensatory systemic responses, including increased blood pressure and heart rate, forming a homeostatic feedback loop that maintains cerebral perfusion. Cerebral autoregulation was historically considered the primary mechanism stabilizing brain blood flow despite systemic pressure changes (Johnson, 1986). However, it operates effectively only within a narrow range and poorly buffers significant drops in cerebral perfusion pressure (Numan et al., 2014; Willie et al., 2014; Brassard et al., 2017).

Baroreceptors in the carotid bifurcation and aortic arch monitor systemic pressure, but their location limits direct sensing of cerebral perfusion (Thrasher, 2005), suggesting the existence of intrinsic brain mechanisms that detect and relay perfusion changes (Marina et al., 2020).

Astrocytes are widely distributed glial cells that sense and modulate neuronal activity (Copeland et al., 2017). They are anatomically well positioned between blood vessels and brain parenchyma to act as intracranial baroreceptors (Iadecola and Nedergaard, 2007). Evidence that higher intracranial pressure increases astrocytic Ca^2+^ levels supports their role as mechanosensors (Marina et al., 2020).

Consistent with their mechanosensory role, astrocytes are equipped with various mechanically activated channels, including pannexin1 hemichannels (Beckel et al., 2014), PIEZO channels (Choi et al., 2015; Velasco-Estevez et al., 2020), and, notably, transient receptor potential vanilloid-4 (TRPV4) channels (Kim et al., 2015; Turovsky et al., 2020; Haidey et al., 2021). Previous work shows that astrocytic detection of vasoconstriction induced by mechanical stimuli is mediated via TRPV4 channels (Kim et al., 2015), which belong to the vanilloid receptor subfamily and are permeable to Ca^2+^ and Mg^2+^ in various tissues (Voets et al., 2002; Watanabe et al., 2003). Astrocyte endfeet with TRPV4 channels encircle pial and parenchymal blood vessels (Benfenati et al., 2011).

The glymphatic pathway has been proposed as a novel convective route for clearing waste from the brain (Iliff et al., 2012; Jessen et al., 2015; Bohr et al., 2022). During sleep, astrocyte shrinkage enlarges perivascular spaces, enhancing flow, whereas wakefulness restricts central nervous system-glymphatic exchange (Iliff et al., 2013). This flow is driven by blood pressure pulses moving in the direction from the arteriole to the venule, generating mechanical forces (including shear stress in narrow extracellular spaces) that astrocytes are likely able to detect (Abbott et al., 2006; Owens et al., 2008; Iliff et al., 2013). Additional pressure gradients arise from rhythmic cerebrospinal fluid fluctuations linked to cardiac cycles, as well as osmotic and volume changes (Mola et al., 2021).

The glymphatic system hypothesis highlights the critical role of polarized aquaporin (AQP4) channels in driving water flux and clearing interstitial solutes from the brain (Xie et al., 2013; Bojarskaite et al., 2024). TRPV4 colocalizes with AQP4, forming a complex essential for control of astrocyte cell volume (Benfenati et al., 2007).

Astrocytes generate L-lactate through glycolytic and glycogenolytic metabolism (Zorec and Vardjan, 2023), generated via glycogen breakdown, regulated by Ca^2+^ and cAMP signaling (Fink et al., 2021; Horvat et al., 2021). Because astrocytes are positioned between the vascular wall and the neural parenchyma, they are exposed to mechanical cues generated by changes in vascular tone, blood flow, intracranial pressure, and perivascular fluid movement (Iadecola and Nedergaard, 2007; Attwell et al., 2010; Kim et al., 2015; Marina et al., 2020; Haidey et al., 2021). The brain has high and continuous energy requirements, but limited local energy reserves (Raichle and Gusnard, 2002); therefore, mechanisms that couple hemodynamic or mechanical cues to astrocytic metabolic responses may contribute to local energy homeostasis (Pellerin and Magistretti, 1994; Attwell et al., 2010; Magistretti and Allaman, 2018). Astrocytes are known to regulate aerobic glycolysis and L-lactate production through Ca^2+^-, cAMP-, glucose-, and glycogen-dependent pathways (Fink et al., 2021; Horvat et al., 2021; Zorec and Vardjan, 2023; Fink et al., 2025). However, whether brief mechanical stimulation is sufficient to trigger intracellular accumulation of L-lactate in astrocytes has not been tested.

We investigated whether mechanical stimulation affects astrocytic metabolism at the single-cell level, specifically focusing on intracellular L-lactate accumulation in isolated primary astrocytes. This approach was designed to identify the astrocytic cell-autonomous component of mechano-metabolic signaling before addressing neuron-dependent consequences in more complex preparations. Although TRPV4 activation by mechanical stress has been linked to cation influx and astrocytic Ca^2+^ signaling (Dunn et al., 2013), whether mechanical activation of astrocytes is coupled to intracellular L-lactate production remains unknown. We hypothesized that mechanical stimulation promotes intracellular L-lactate accumulation, highlighting astrocytic mechano-metabolic transduction as a mechanism for adapting to mechanical cues. Our results show that a brief mechanical pulse triggers a sustained increase in L-lactate, supporting the concept of astrocytes as mechano-metabolic integrators in the brain.

## 2 Materials and methods

### 2.1 Study approval

All experimental procedures involving animals were performed in compliance with the International Guiding Principles for Biomedical Research Involving Animals, established by the Council for International Organisations of Medical Sciences, and the Animal Protection Act (Official Gazette of the RS, No. 38/13, consolidated texts 21/18, 92/20, 159/21). The study protocols were approved by the Administration for Food Safety, Veterinary Sector and Plant Protection (Republic of Slovenia, Ministry of Agriculture, Forestry and Food, Dunajska cesta 22, 1,000 Ljubljana), under permit numbers U34401-27/2020/6, U34401-26/2020/4, U34401-26/2020/7, U34401-27/2025/4, and U34401-26/2025/6.

### 2.2 Cell cultures and plasmid transfection

Primary astrocyte cultures were prepared from the cerebral cortices of neonatal (postnatal days 2–3) male and female Wistar Han outbred rats (RccHan: WIST; RRID: RGD_149735906; Inotiv, the Netherlands). Animals were rapidly decapitated without prior anesthesia, in accordance with EU Directive 2010/63/EU on animal welfare, as previously described (Schwarts and Wilson, 1992). Astrocyte cultures were purified from other brain cell types by repeated overnight shaking at 225 rpm at room temperature, performed three times upon reaching confluency. Cultures were maintained in high-glucose Dulbecco’s modified Eagle’s medium (Sigma-Aldrich, St. Louis, MO, USA, Cat. No. D6546), supplemented with 10% fetal bovine serum (Sigma-Aldrich, Cat. No. F-7524), 1 mM sodium pyruvate (Sigma-Aldrich, Cat. No. S-8636), 2 mM L-glutamine (Sigma-Aldrich, Cat. No. G-3126), and 25 μg/mL penicillin–streptomycin (Sigma-Aldrich, Cat. No. P-0781), in a humidified incubator (95% air, 5% CO_2_). The medium was replaced every 2 days.

For experiments, purified astrocytes were detached using trypsin–EDTA (Sigma-Aldrich, Cat. No. T-3924) and seeded onto poly-L-lysine-coated coverslips (Sigma-Aldrich, Cat. No. P-1524). Cultured cells were maintained in complete growth medium until experimentation.

Cells were transfected with a genetically encoded FRET-based lactate nanosensor (San Martín et al., 2013) Laconic (Addgene, Watertown, MA, USA; Plasmid Cat. No. 44238; RRID: Addgene_44,238), at least 16 h before experimentation. Transfection was performed using FuGENE 6 Transfection Reagent (Promega, Madison, WI, USA, Cat. No. E-2692) according to the manufacturer’s instructions in an antibiotic- and serum-free lipofection medium.

### 2.3 Experimental extracellular solutions

The extracellular solutions contained 135.3 mM NaCl (Sigma-Aldrich, Cat. No. S-7653), 5 mM KCl (Sigma-Aldrich, Cat. No. P3911), 10 mM HEPES (4-(2-hydroxyethyl)-1-piperazineethanesulfonic acid) (Sigma-Aldrich, Cat. No. H-3375), 0.5 mM NaH_2_PO_4_·H_2_O (Sigma-Aldrich, Cat. No. S0751), 5 mM NaHCO_3_ (Sigma-Aldrich, Cat. No. S-5761), 2 mM MgCl_2_ (Sigma-Aldrich, Cat. No. M-8266), 1.8 mM CaCl_2_ (Sigma-Aldrich, Cat. No. 21115), and 3 mM D-glucose (Sigma-Aldrich, Cat. No. G8279); pH adjusted (S20 SevenEasy, Mettler Toledo, Columbus, OH, USA) to 7.2 with NaOH (Merck, Darmstadt, Germany, Cat. No. 1064981000). Osmolarity ranged from 290 to 310 mOsm, measured with a freezing-point osmometer (Osmomat030; Gonotech GmbH, Berlin, Germany).

The extracellular solution for a positive control consisted of 95.3 mM NaCl, 5 mM KCl, 10.0 mM HEPES, 0.5 mM NaH_2_PO_4_·H_2_O, 5.0 mM NaHCO_3_, 2.0 mM MgCl_2_, 1.8 mM CaCl_2_, 3.0 mM D-glucose, and 20.0 mM L-lactate (Sigma-Aldrich, Cat. No. 71718); pH adjusted to 7.2 with NaOH. Osmolarity ranged from 290 to 310 mOsm, measured with a freezing-point osmometer.

For monitoring changes in intracellular L-lactate after mechanical stimuli and assessing the involvement of TRPV4 channels, two different 10 μM TRPV4 antagonist solutions were used: HC-067047 (2-methyl-1-(3-morpholinopropyl)-5-phenyl-N-(3-(trifluoromethyl) phenyl) -1H-pyrrole-3-carboxamide; Sigma-Aldrich, Cat. No. 616521) or RN-1734 (2,4-dichloro-N-isopropyl-N-(2-isopropylaminoethyl)benzenesulfonamide; Sigma-Aldrich, Cat. No. 616520). Stock solutions of antagonists were prepared at 50 mM in dimethyl sulfoxide (DMSO) and diluted into extracellular solution to achieve a final working concentration of 10 μM. The final DMSO concentration applied to the cells did not exceed 0.02%.

### 2.4 Immunocytochemistry

Cultured astrocytes were immunolabeled with rabbit polyclonal anti-TRPV4 antibody (Alomone Labs, Jerusalem, Israel, Cat. No. ACC-034) diluted 1:200 and mouse monoclonal anti-GFAP antibody (Sigma-Aldrich, Cat. No. G3893) diluted 1:800. Cells were first washed with phosphate-buffered saline (PBS; Sigma-Aldrich, Cat. No. P4417), then fixed and permeabilized in 4% formaldehyde solution (Thermo Fisher Scientific, Waltham, MA, USA, Cat. No. 28908) for 15 min at room temperature (22 °C–25 °C). After fixation, astrocytes were incubated in blocking buffer consisting of 3% bovine serum albumin (Sigma-Aldrich, Cat. No. A2153) and 10% goat serum (Sigma-Aldrich, Cat. No. G-6767) in PBS for 1 h at 37 °C to reduce non-specific staining. Primary antibody labeling was conducted overnight at 4 °C. After thorough washing with PBS, cells were incubated for 45 min at 37 °C with goat polyclonal anti-rabbit IgG conjugated to Alexa Fluor 488 (Invitrogen, Waltham, MA, USA, Cat. No. A-11008) at 1:600 to detect TRPV4, and goat anti-mouse IgG conjugated to Alexa Fluor 546 (Invitrogen, Cat. No. A-11003) at 1:600 to detect GFAP. Slides were mounted using Slowfade Gold antifade mountant with DAPI (Invitrogen, Cat. No. S36942) and imaged using a confocal microscope (Zeiss LSM700 AxioObserver; Plan Apochromat 63×/1.4 NA oil immersion lens) with excitation via diode laser at 405 nm for DAPI, 488 nm for Alexa Fluor 488/TRPV4, and 555 nm for Alexa Fluor 546/GFAP. Control experiments using secondary antibodies without primary antibodies produced no detectable fluorescent signal, thus confirming the specificity of the secondary antibodies’ immunolabeling.

### 2.5 Mechanical stimulation

Mechanical stimuli were applied using a glass patch-clamp micropipette mounted on a micromanipulator (Eppendorf InjectMan 4, Eppendorf, Hamburg, Germany) and connected to a microinjection system (FemtoJet 4i, Eppendorf), which allowed precise control of the applied pressure. The glass micropipette (standard-walled borosilicate glass, 30–0058, Harvard Apparatus, Holliston, MA, USA), filled with extracellular solution, was positioned approximately 10 μm from the targeted astrocyte at the center of the field of view (Supplementary Figure S2). Stimuli were applied for 1.5 s by delivering a set volume of extracellular solution. Baseline FRET measurements with the Laconic nanosensor were acquired before stimulation with either a single pressure pulse of a given magnitude (5, 50, 500, and 1,000 hPa) or a series of stepwise pressure pulses with increasing magnitudes (5, 50, 500, and 1,000 hPa), each lasting 1.5 s. These values should not be interpreted as uniform hydrostatic pressures acting on the entire cell or as direct equivalents of intracranial or intravascular pressure. The actual mechanical stress at the cell surface depends on tip geometry and cell compliance. Dose–response curves were generated for both stimulation regimes. Two stimulation regimes were used to probe different temporal patterns of astrocytic mechano-metabolic responsiveness. The single 1.5-s pressure pulse was used as a minimal brief perturbation, conceptually related to short mechanical events that may accompany transient activity-induced vascular responses. The sequential pressure-pulse protocol was not designed to represent continuous pressure elevation; rather, it was used to test whether repeated, graded mechanical events applied to the same astrocyte produce cumulative or state-dependent changes in intracellular lactate. Thus, the pressure values reported here should be interpreted as controlled micropipette command pressures rather than direct reproductions of vascular pressure waveforms *in vivo*. In addition, mechanical stimulation by bolus addition of 200 μL was performed using a pipette in astrocytes preincubated with TRPV4 antagonists (HC-067047 or RN-1734) to assess the specific involvement of TRPV4 channels in the modulation of intracellular lactate. Pharmacological experiments were performed using bolus addition rather than the sequential micropipette pressure-pulse protocol. This design allowed mechanical stimulation to be applied with the same antagonist-containing solution used during preincubation and avoided cumulative effects of repeated mechanical stimulation during pharmacological testing.

### 2.6 FRET measurements and data analysis

FRET microscopy, which monitors energy transfer between fluorescent proteins within genetically encoded nanosensors, was used to measure [lactate]_i_ in single astrocytes in real time. Laconic is a genetically encoded FRET-based L-lactate nanosensor composed of the bacterial lactate-binding transcription factor LldR positioned between the fluorescent proteins mTFP and Venus. The binding of L-lactate induces a conformational change that decreases FRET efficiency and increases the mTFP/Venus emission ratio (San Martín et al., 2013). Therefore, changes in the mTFP/Venus ratio were used as a ratiometric readout of relative changes in intracellular L-lactate concentration. This ratiometric approach reduces, but does not eliminate, sensitivity to differences in sensor expression, illumination intensity, and cell thickness. Data are reported as normalized relative changes in FRET ratio rather than absolute intracellular L-lactate concentrations.

Changes in nanosensor fluorescence ratios (ΔFRET) for monomeric teal fluorescent protein (mTFP)/Venus (Laconic) were recorded using a fluorescence microscope (Zeiss Axio Observer. A1; Zeiss, Oberkochen, Germany) equipped with a C-Apochromat 63×/1.2 NA water immersion objective (Zeiss) and a digital camera (Axiocam 702 mono, Zeiss). Nanosensors were excited at 430 nm using a Colibri 7 LED module (Zeiss). Emitted fluorescence was separated into cyan (460 nm) and yellow (520 nm) channels by an image splitter (Photometrics DV2; Optical Insights, Tucson, AZ, USA). Images were captured every 10 s with an exposure time of 1,000 ms using Zeiss Zen software. Therefore, kinetic parameters occurring on a timescale shorter than one acquisition interval could not be resolved with high temporal precision. For the sequential stimulation protocol, the fall half-time after transient spikes was calculated as an apparent value by interpolation between the two sampled points that bracket the half-amplitude level. Because crossings occurring within the first post-peak sampling interval are not directly observed, apparent fall half-times near 5 s represent the practical lower temporal limit of this analysis. Values at or below this limit were interpreted as resolution-limited and were not used to infer precise sub-10-s kinetics. Data analysis was performed using Microsoft Excel and custom MATLAB scripts. Figures were assembled using GraphPad Prism 10.5.

Before the experiment, cells were incubated for 30 min in extracellular solution. After baseline measurements of the FRET signal for 300 s, cells were exposed to a mechanical stimulus – a pressure pulse, as described above. In pharmacologic experiments, cells were incubated for 30 min in a solution containing an antagonist (10 μM, HC-067047 or RN-1734), and after the 300-s baseline measurement, cells were exposed to manual addition of 200 μL of the same solution (10 μM HC-067047 or RN-1734). At 900 s, cell viability and sensor functionality were validated by adding 20 mM L-lactate.

Changes in the mTFP/Venus fluorescence ratio within a region of interest were measured by manually outlining the cell, excluding the nucleus, and subtracting the background fluorescence from both signals. Ratio traces were corrected for photobleaching-related baseline drift using a custom MATLAB routine (fit of the initial steady-state segment extrapolated over the recording and subtracted). An increase in the mTFP/Venus ratio reflects an increase in intracellular L-lactate. Weakly fluorescent cells displayed undetectable responses; therefore, recordings were excluded if the mTFP fluorescence after background subtraction was less than half the average background fluorescence. The amplitude calculation and data normalization are described in the Results section for each figure separately.

Known limitations of Laconic include semi-quantitative interpretation without cell-specific calibration, potential sensitivity to intracellular pH changes, and the sensor’s reporting intracellular rather than extracellular lactate. Therefore, the present data are interpreted as relative changes in cytosolic lactate signal (San Martín et al., 2013).

### 2.7 Quantification and statistical analysis

Unless otherwise stated, all results are presented as means ± SEM. Statistical significance for comparisons between two samples was determined using Student’s two-tailed t tests or Mann–Whitney U test on ranks, where normality (Shapiro–Wilk) or equal variance tests failed. For comparisons involving more than two samples, one-way ANOVA, Friedman test (nonparametric repeated measures), or Kruskal–Wallis test was used, as appropriate. Statistical calculations and final images were generated using SigmaPlot 11.0, Microsoft Excel, GraphPad Prism 10.5, and Python software. Differences were considered significant if *p* < 0.05, *p* < 0.01, and *p* < 0.001, and are indicated with one, two, or three asterisks, respectively. In the box plots, the box represents the interquartile range, containing the middle 50% of the data. A black line within the box indicates the median, the mean is indicated by +, and the whiskers represent the 10th and 90th percentiles. Sample sizes are reported next to the boxes. df, degrees of freedom; F, the F-statistic used in one-way ANOVA or the Friedman test; H, the H-statistic used in the Kruskal–Wallis test. Q, q, and t signify the test statistic used in the *post hoc* analyses. The sample size was not predetermined using statistical methods, and blinding, randomization, and outlier tests were not performed.

## 3 Results

### 3.1 TRPV4 channel immunoreactivity was detected in purified primary rat cortical astrocyte cultures

**Figure 1:**
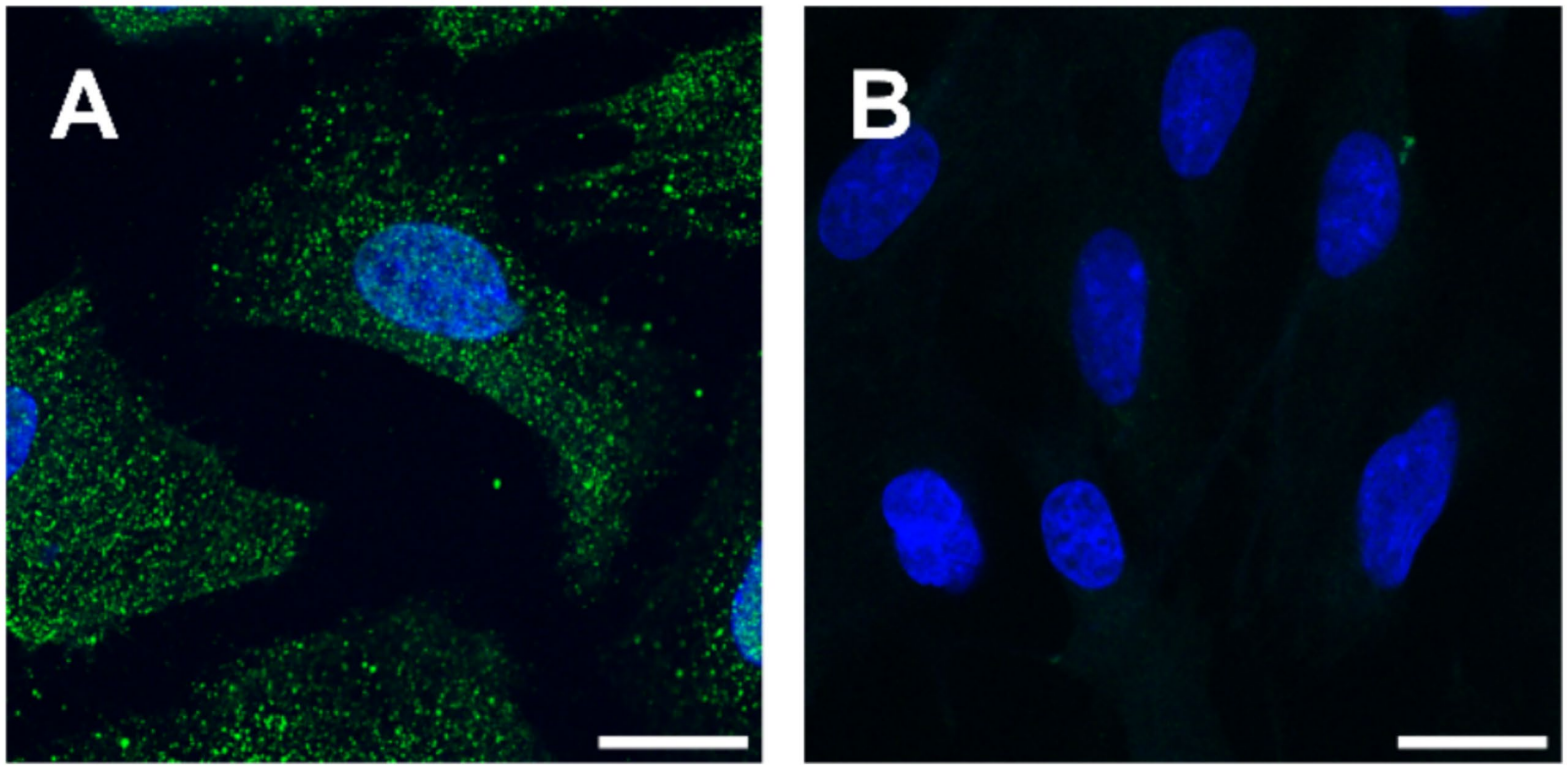
Localization of TRPV4 channels in primary rat astrocytes. **(A)** Immunofluorescence staining shows TRPV4 channel expression on astrocytes (green). **(B)** A control experiment without primary antibodies demonstrates the absence of non-specific staining. Nuclei are counterstained with DAPI (blue). Scale bar: 20 μm.

To assess whether cells in our purified primary rat cortical astrocyte cultures exhibit TRPV4 immunoreactivity, we immunolabeled the cultures with an anti-TRPV4 antibody and performed confocal microscopy. This approach confirmed apparent TRPV4 immunoreactivity in astrocytes (Figure 1A; green fluorescence). The labeling appears specific, as demonstrated by control experiments in which primary antibodies were omitted and showed no detectable fluorescent signal (Figure 1B). Additional TRPV4/GFAP co-labeling is shown in Supplementary Figure S1.

### 3.2 Dose-dependent increase in [lactate]_i_ after mechanical stimulation

**Figure 2:**
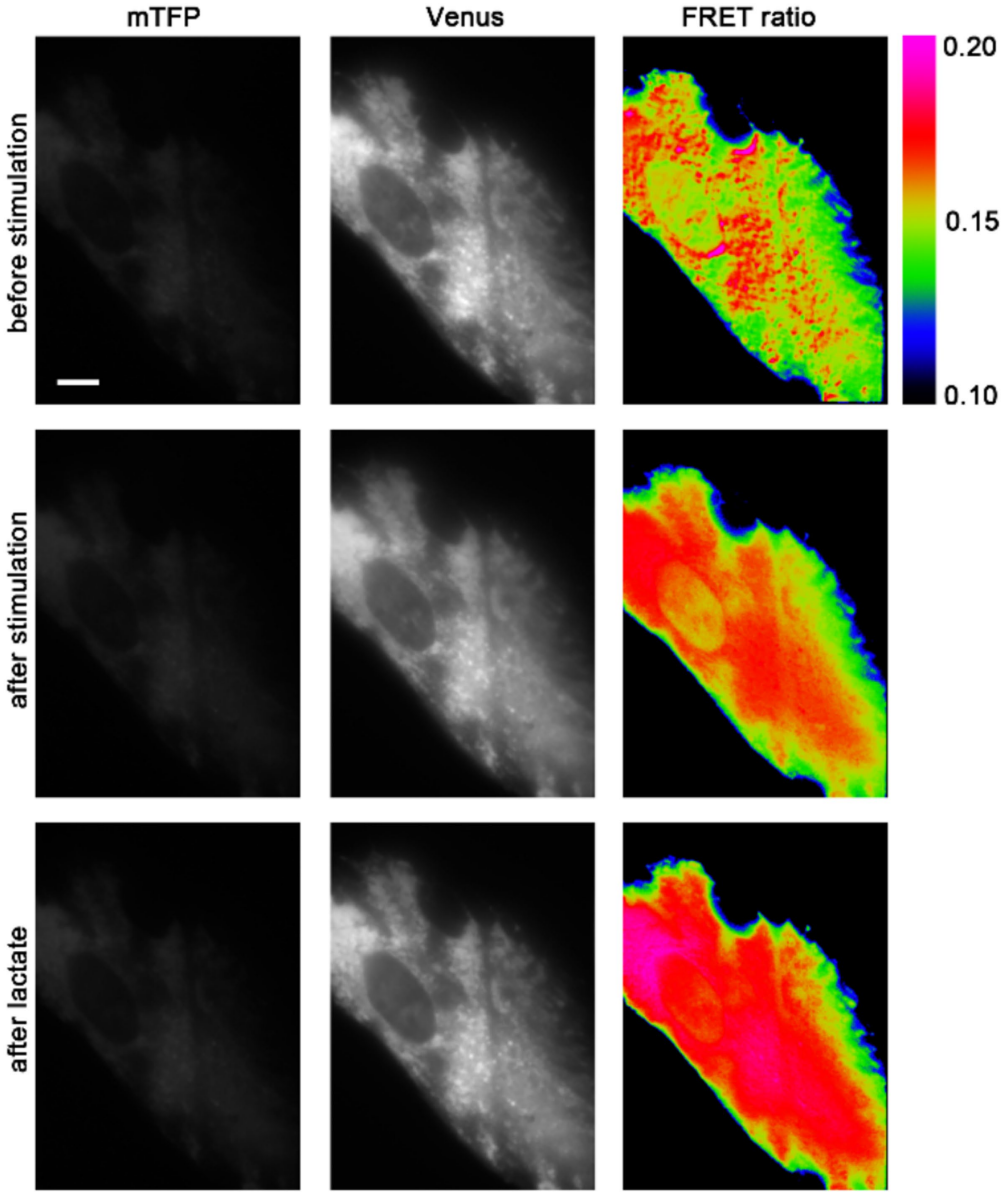
Lactate-dependent changes in the mTFP/Venus FRET ratio in cultured astrocytes expressing Laconic. Representative fluorescence images of a cultured astrocyte expressing the genetically encoded lactate nanosensor Laconic. The left and middle columns show donor mTFP and acceptor Venus emission, respectively, and the right column shows the corresponding pixel-wise pseudocolour mTFP/Venus FRET ratio from the same cell. Images are shown before stimulation, 600 s after 500 hPa stimulation, and 100 s after application of 20 mM lactate as a positive control. Stimulation increased the FRET ratio, with a further increase following lactate application, consistent with an elevation of intracellular lactate. The pseudocolor scale represents the FRET ratio from 0.10 to 0.20, with blue indicating lower values and pink indicating higher values. Scale bar: 10 μm.

In all experiments investigating mechanotransduction, we utilized a precisely controlled mechanical stimulation system, detailed in the Materials and Methods section. Briefly, a glass micropipette, typically used for electrophysiologic patch-clamp measurements, was secured in a micropipette holder, which was connected to a micromanipulator and microinjector. The micropipette was filled with extracellular solution and positioned approximately 10 μm from the target astrocyte, centered in the field of view (Supplementary Figure S2). Micropipettes filled with the extracellular solution were used to apply pressure pulses of 1.5 s duration to the cells. Representative mTFP, Venus, and mTFP/Venus ratio images are shown in Figure 2 to illustrate sensor expression, cytosolic localization, and the ratiometric response to mechanical stimulation.

**Figure 3:**
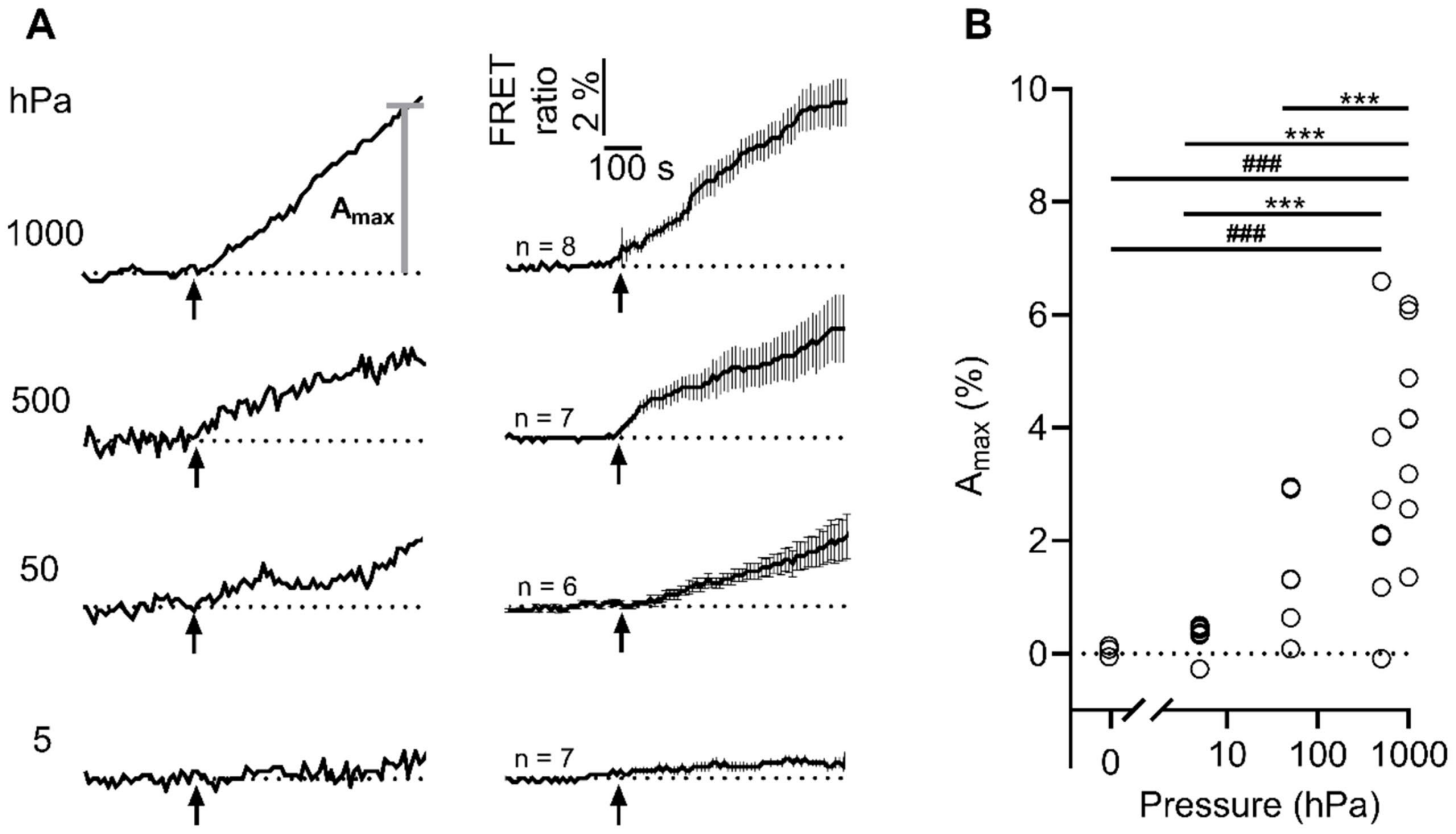
Dose-dependent increase in [lactate]_i_ after a single pressure pulse mechanical stimulation. **(A)** Representative recordings (left panel) and mean ± SEM normalized time courses of the FRET ratio (right panel) after single pulses (arrows) of mechanical stimulation at the specified pressures (duration 1.5 s) applied at the times indicated by the arrows. The A_max_ designation represents the calculated amplitude differences, determined using baseline values 50 s before stimulation and mean maximal amplitude values. The dotted horizontal lines represent the baseline value of 0% of the normalized FRET ratio. **(B)** Mean maximal amplitudes of [lactate]_i_ increase after mechanical stimulation at pressures of 0 hPa (*n* = 3), 5 hPa (*n* = 7), 50 hPa (*n* = 6), 500 hPa (*n* = 7), and 1,000 hPa (*n* = 8). Each circle indicates data obtained from a single cell. Experiments were performed on cells isolated from at least three different animals. Statistical significance was determined using one-way ANOVA followed by Holm-Sidak *post hoc* analysis (***p < 0.001, ###*p* < 0.001 versus no stimulation).

To investigate the dynamics of intracellular L-lactate concentration ([lactate]_i_) in astrocytes after pressure-induced mechanical stimulation, we used the Förster resonance energy transfer (FRET) method with the Laconic genetically encoded lactate nanosensor (San Martín et al., 2013). Astrocytes were stimulated with a single pressure pulse, indicated by a black arrow (Figure 3A), at various pressures (5, 50, 500, or 1,000 hPa). A baseline recording of 300 s preceded the stimulation, followed by 600 s of post-stimulation monitoring. Nanosensor functionality was then validated by adding 20 mM L-lactate and observing the response for an additional 300 s (Figure 2).

Application of a brief 1.5-s pressure pulse resulted in a sustained and significant increase in the FRET ratio, reflecting an increase in [lactate]_i_. Specifically, stimulation at pressures of 5, 50, 500, and 1,000 hPa resulted in mean increases in the FRET ratio of 0.33% ± 0.10% (*n* = 7), 1.54% ± 0.48% (*n* = 6), 2.63% ± 0.81% (*n* = 7), and 4.07% ± 0.59% (*n* = 8), respectively. No significant change from baseline was observed at 0 hPa (0.05% ± 0.06%, *n* = 3). Data were obtained with cells isolated from at least three animals per experimental condition. Mean maximal amplitudes for increases in [lactate]_i_ were calculated as an average of the signal over a 50-s period, where the signal reached maximum (indicated by the gray line labeled A_max_ in Figure 3A), and compared with the baseline average values obtained before stimulation (a 50-s epoch before the stimulus).

Statistically significant differences were identified at the highest pressures tested (1,000 hPa and 500 hPa; 4.07% ± 0.59 and 2.63% ± 0.81%) compared with the control group of 0 hPa, 0.05% ± 0.06% (one-way analysis of variance [ANOVA], *p* < 0.001, *F* = 8.29, df = 4), followed by Holm-Sidak *post hoc* tests (###*p* < 0.001; *t*_(0 vs 1,000 hPa)_ = 4.11, *t*_(0 vs 500 hPa)_ = 2.59). Moreover, statistically significant differences were also identified at the highest pressures tested (500 and 1,000 hPa) compared with the lower pressures of 5 and 50 hPa (0.33% ± 0.10, and 1.54% ± 0.48%; one-way ANOVA, *p* < 0.001, *F* = 8.31, df = 3), followed by Holm-Sidak *post hoc* tests (***p < 0.001; *t*_(5 vs 500 hPa)_ = 2.87, *t*_(5 vs 1,000 hPa)_ = 4.81, *t*_(50 vs 1,000 hPa)_ = 3.12).

These results demonstrate a dose-dependent relationship, whereby increasing the applied pressure pulse increases [lactate]_i_. Moreover, a brief single pressure-pulse induces a sustained increase in [lactate]_i_.

### 3.3 Sequential pressure-pulse applications enhance [lactate]_i_ increase in astrocytes

**Figure 4:**
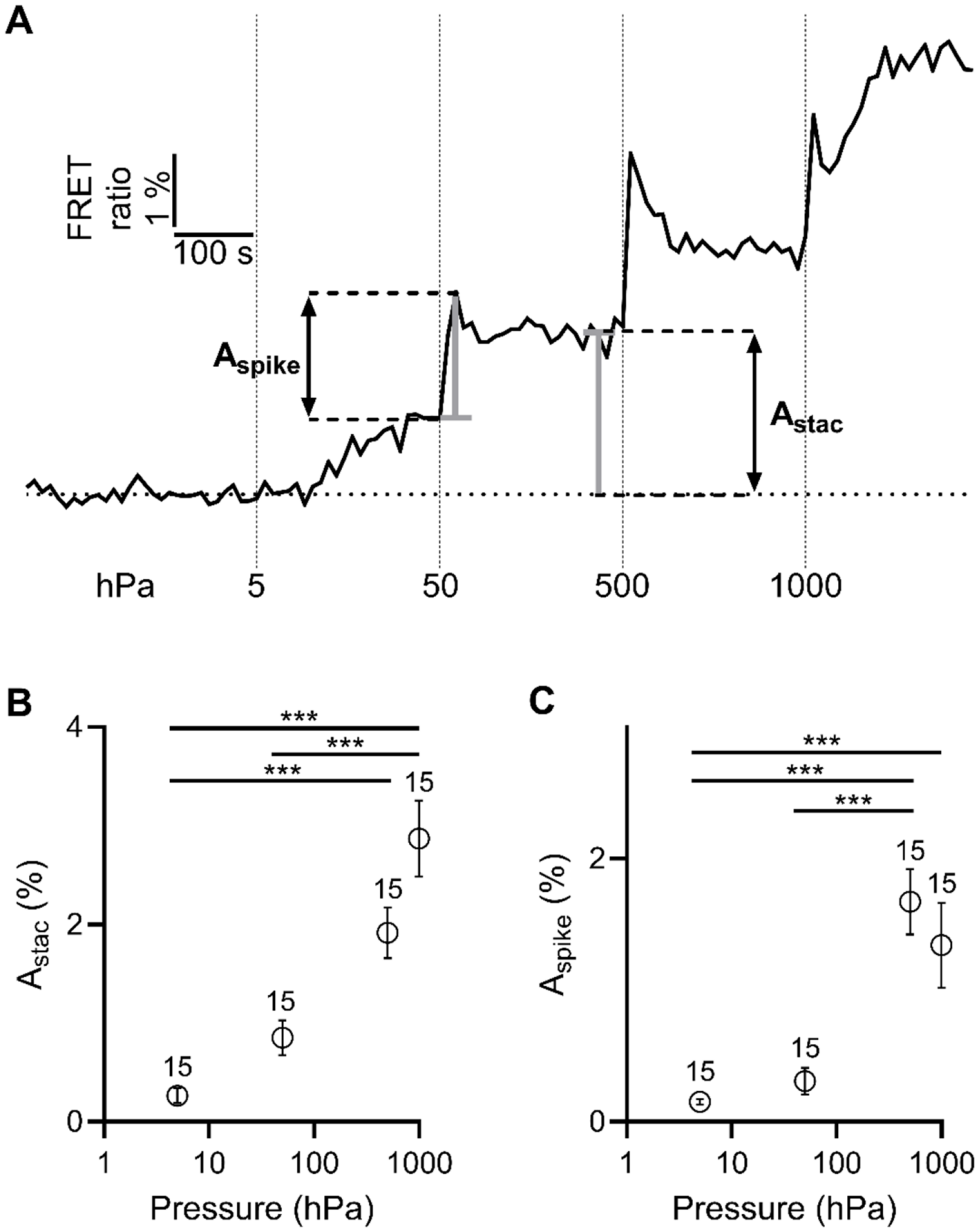
Increase in [lactate]_i_ in astrocytes exposed to sequential application of pressure pulses. **(A)** Representative recording illustrating the effects of sequential mechanical stimulations consisting of pressure pulses delivered every 230 s with increasing pressures, indicated with dotted vertical lines. A_stac_ denotes change in the stationary-state signal amplitude (calculated from baseline values within a 50-s period before the initial stimulus to the mean stationary-state values within a 50 s period after each stimulus). A_spike_ indicates transient changes in amplitude calculated from values within a 50-s period immediately preceding each stimulus to peak values observed within 50 s post stimulus. The dotted horizontal line represents the baseline value of 0% of the normalized FRET ratio. **(B)** Plots of mean ± SEM A_stac_ amplitudes for [lactate]_i_ responses to sequential stimuli at 5, 50, 500, and 1,000 hPa (*n* = 15 cells tested). **(C)** Plots of mean ± SEM A_spike_ amplitudes for transient [lactate]_i_ increases after sequential stimuli at 5, 50, 500, and 1,000 hPa (*n* = 15 cells tested). Data were derived from cells isolated from at least three animals. Statistical significance was assessed using Friedman repeated measures ANOVA on ranks followed by Tukey’s *post hoc* test (B; ***p ≤ 0.001 and C; ***p ≤ 0.001).

We tested whether the sequentially increasing pressure pulses applied to the same cell affect [lactate]_i_. After a 300-s baseline recording, individual astrocytes were sequentially stimulated with increasing pressure pulses (5, 50, 500, and 1,000 hPa) at 230-s intervals (see vertical dotted lines in Figure 4A). The sequential protocol should therefore be interpreted as a test of cumulative mechanometabolic responsiveness rather than as a model of a single persistent pressure stimulus. We computed two sets of amplitudes to characterize the responses. The first set, labeled A_stac_ in Figure 4A and presented in Figure 4B, represents sustained amplitude increases calculated using initial baseline values before the first stimulus and the mean plateau values after each stimulus (measured before the subsequent stimulus). The second set, labeled A_spike_ in Figure 4A and depicted in Figure 4C, represents transient amplitude increases calculated using values immediately preceding each stimulus and the maximal response values recorded within 50 s post stimulation. Each amplitude value used for the calculations represents the average value within a 50-s window, except for A_spike_.

Pressure-pulse application (5 hPa, *n* = 15) typically resulted in a monotonic, exponential-like increase in [lactate]_i_. The successive pressure pulses, set at higher pressures (50, 500, and 1,000 hPa, *n* = 15 per group), elicited a swift transient increase to a peak (labeled A_spike_, Figure 4A), which then exponentially decayed to a stationary-state value that remained higher than the value observed before the pulse was applied (labeled A_stac_, Figure 4A). To quantify the data, we plotted the amplitudes of A_stac_ and A_spike_ as a function of the pressure-pulse magnitude (Figure 4B), determined as a percentage relative to the basal level and the pre-stimulus level, respectively. Each A_stac_ amplitude represents the signal average of a 50-s epoch, except for A_spike_, where only the maximum value within the 50-s epoch was used.

The initial 5 hPa stimulus elicited responses comparable with single-stimulus experiments (Figure 3) with an increase in the amplitude A_stac_ of [lactate]_i_ 0.26% ± 0.07%, significantly different from zero (one-sample *t* test). Increasing the pressure to 50 hPa and beyond produced transient spikes (A_spike_), followed by increased steady-state responses (A_stac_). Subsequent higher-intensity stimuli of 50, 500, and 1,000 hPa resulted in stationary-state increases in A_stac_ (Figure 4B) of 0.85% ± 0.18, 1.92% ± 0.26, and 2.87% ± 0.39%, respectively; only A_stac_ at 1000 hPa and 500 hPa was significantly higher than A_stac_ at 5 hPa and 50 hPa. Higher-intensity stimuli of 500 and 1,000 hPa resulted in transient spike amplitudes A_spike_ of 1.67% ± 0.25 and 1.34% ± 0.32%, respectively, with A_spike_ at 500 and 1,000 hPa stimulation significantly higher than A_spike_ observed at 5 hPa and 50 hPa (0.15% ± 0.02 and 0.31% ± 0.10%), normalized to the baseline values.

Differences in stationary-state responses were statistically significant at 5 hPa compared with 500 and 1,000 hPa stimuli, as well as between 50 hPa and 1,000 hPa stimuli; the transient spikes exhibited significant differences between 5 hPa and both 500 and 1,000 hPa stimuli, as well as between 50 hPa and 500 hPa; other comparisons were not statistically significant (Friedman repeated measures ANOVA on ranks for A_stac_) (***p ≤ 0.001; *χ*^2^ = 45, df = 3) followed by Tukey’s *post hoc* analysis (***p < 0.001; *q*_(1,000 hPa vs 5 hPa)_ = 9, *q*_(500 hPa vs 5 hPa)_ = 6, *q*_(1,000 hPa vs 50 hPa)_ = 6); Friedman repeated measures ANOVA on ranks for A_spike_ (***p ≤ 0.001; *χ*^2^ = 25.64, df = 3) followed by Tukey’s *post hoc* test (***p ≤ 0.001; *q*_(1,000 hPa vs 5 hPa)_ = 4.6, *q*_(500 hPa vs 5 hPa)_ = 6.4, *q*_(500 hPa vs 50 hPa)_ = 5).

These results revealed that astrocytes display diverse responses to mechanical stimulation, prompting us to investigate whether these distinct responses exhibit different mechanosensitivities.

### 3.4 Mechanical sensitivity of astrocytes

**Figure 5:**
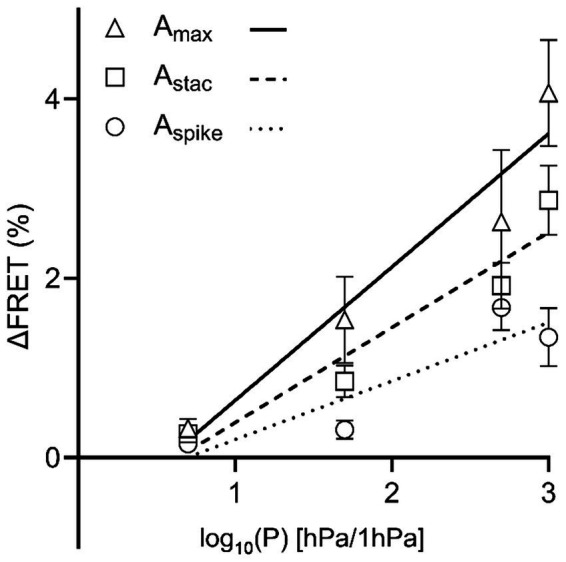
Mechanical sensitivity of astrocytes. The plots showing changes in [lactate]_i_ as ΔFRET (%) for the three datasets of mechanical stimulation (A_max_, A_stac_, A_spike_) after mechanical stimulation at 5, 50, 500, and 1,000 hPa, with corresponding lines obtained by linear regression fits. *P* denotes pressure. Mechanical stimulation, whether delivered as a single pressure pulse or a sequence of multiple pressure pulses, appears to activate a similar astrocytic response mechanism. No statistically significant differences between the slopes of these lines were detected (Kruskal–Wallis; *p* = 0.163).

With three distinct datasets of amplitudes representing an increase in [lactate]_i_ after mechanical stimulation with pressure pulses of 5, 50, 500 or 1,000 hPa, derived from either a single pulse-induced stimulation (Figure 3B, circles A_max_) or sequential application of multiple pressure pulses to a single cell (Figure 4, A_spike_, representing peak amplitudes of transient signal increases, and A_stac_, amplitudes of stationary-state signal changes), we explored whether the mechanosensitivities were distinct. Figure 5 plots changes in FRET ratios obtained from the distinct datasets, marked with different symbols, as a function of the applied pressure (hPa). We fitted a linear function of the form shown in Equation (1):


ΔFRET[%]=klog10(P[hPa/1hPa])+ΔFREToffset (1)

Where ΔFRET [%] is the normalized change in the Laconic FRET ratio (percentage of baseline), *k* is the log-pressure sensitivity (slope, percentage per decade), and ΔFRET_offset_ is the ordinate intercept of the fit, i.e., the value of ΔFRET at *p* = 1 hPa. Regression parameters are shown in Table 1.

**Table 1:** Linear regression parameters for each mechanical stimulation dataset (A_max_, A_stac_, A_spike_), including statistical details and calculated extrapolated thresholds (*P*_th_) of mechanical sensitivity.

Dataset	*k*, mean ± SEM (*n*)	ΔFRET_offset_, mean ± SEM	*p* value	log_10_(*P*_th_) ± SEM; *P*_th_ (+SEM/−SEM)
A_max_	1.50 ± 0.31 (28)	−0.85 ± 0.70	<0.0001	0.57 ± 0.47 log_10_(hPa/1 hPa); 3.7 (+7.3/−2.5) hPa
A_stac_	1.06 ± 0.14 (60)	−0.67 ± 0.32	<0.0001	0.63 ± 0.30 log_10_(hPa/1 hPa); 4.3 (+4.3/−2.1) hPa
A_spike_	0.65 ± 0.12 (60)	−0.45 ± 0.27	0.0005	0.70 ± 0.41 log_10_(hPa/1 hPa); 5.0 (+7.8/−3.0) hPa

ΔFRET [%] = k log_10_(P[hPa/1 hPa]) + ΔFRET_offset_.

The results revealed that distinct mechanical stimulation protocols, whether delivered as a single pressure pulse or as a sequence of multiple pressure pulses to a single cell, all show a similar mechanosensitivity in the range of 0.65 to 1.50 [log_10_(hPa/1 hPa)], with slopes not significantly different (Kruskal–Wallis test, *p* = 0.163; *H* = 3.62, df = 2), indicating a similar underlying astrocyte response mechanism.

Given that all forms of mechanical stimulation evoked measurable changes in [lactate]_i_, we defined the threshold pressure (*P*_th_) as the minimal pressure that initiates a response. Operationally, we obtained this threshold as the abscissa intercept of the linear fit in log-pressure coordinates, i.e., the value of log_10_(*P*_th_/1 hPa) at which ΔFRET returns to baseline. This abscissa crossing is reported as the log quantity, and the corresponding calculated pressures derived using log-transformed pressure values (Table 1) were 3.7 hPa (A_max_ dataset), 4.3 hPa (A_stac_ dataset), and 5.0 hPa (A_spike_ dataset).

### 3.5 Kinetics of pressure-pulse-induced responses in [lactate]_i_

**Figure 6:**
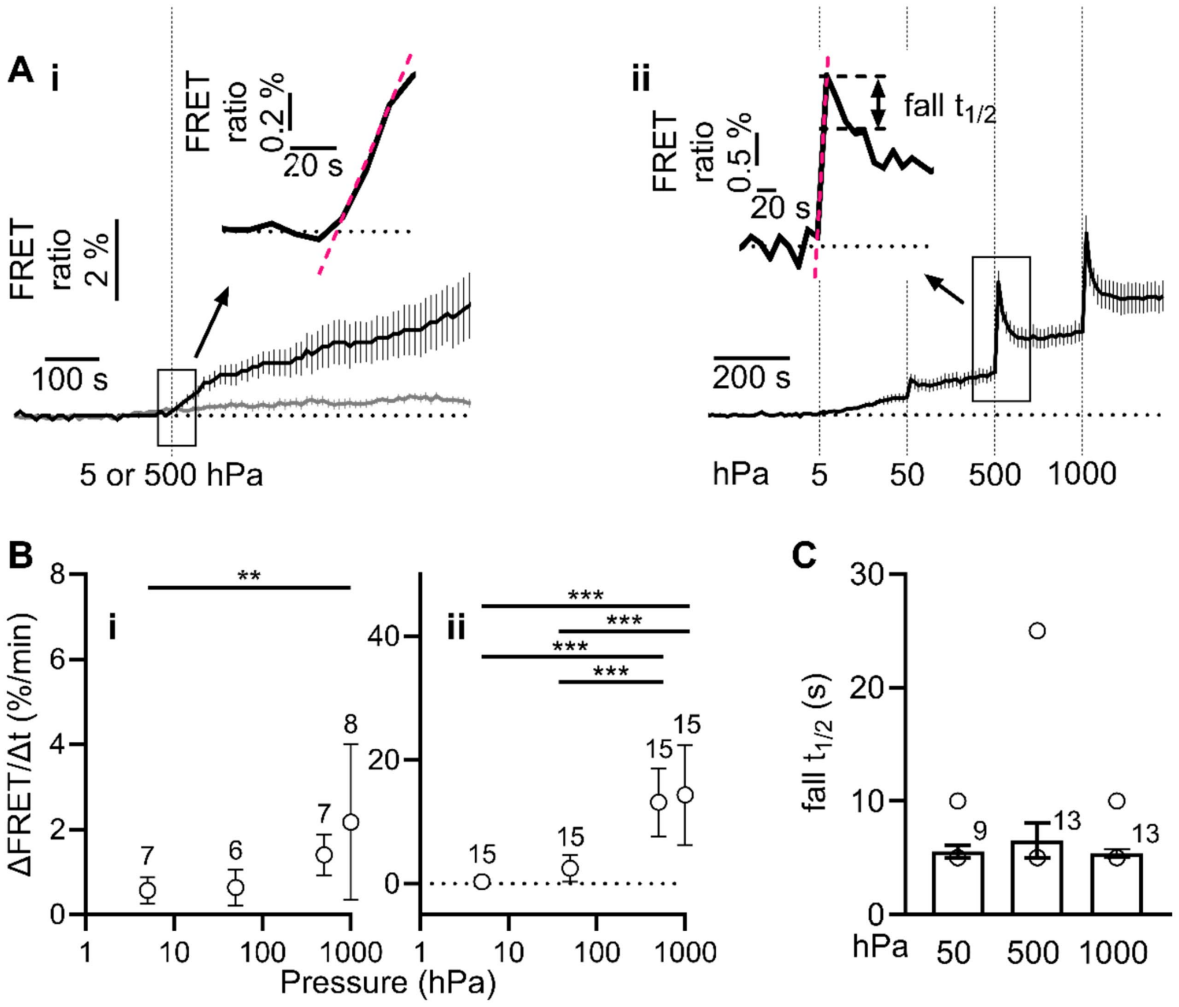
Kinetics of pressure-pulse-induced responses in [lactate]_i_ **(Ai)** Mean ± SEM normalized time courses of the FRET ratio after single mechanical stimulations with 5 hPa (gray line, *n* = 7) and 500 hPa (black line, *n* = 7), respectively. The inset shows a representative recording at 500 hPa illustrating the initial rate of increase in intracellular lactate ([lactate]_i_), indicated by a red dashed line. **(Aii)** Mean ± SEM normalized time courses of the FRET ratio after sequential mechanical stimulations (*n* = 15). The enlarged inset shows a representative recording at 500 hPa, highlighting the initial rate of increase in [lactate]i (red dashed line) and demonstrating the calculation of the half fall time (*t*_1/2_) after maximal increase in lactate. **(Bi)** Maximal rates of the initial increase in [lactate]_i_ after single stimulations, calculated using the steepest 20-s interval within the first 200 s post stimulus, at 5 (*n* = 7), 50 (*n* = 6), 500 (*n* = 7), and 1,000 hPa (*n* = 8). **(Bii)** Initial rates after sequential stimulations, determined from maximal increases within the first 10 s post stimulus, at pressures of 5, 50, 500, and 1,000 hPa (*n* = 15 per group). **(C)** Apparent fall time *t*_1/2_ ± SEM for [lactate]_i_ normalization after the peak response, calculated as the time required for [lactate]_i_ to return halfway from the transient spike maximum toward the subsequent stationary level, using a 50-s averaging window post stimulus, at pressures of 50 (*n* = 9), 500 (*n* = 13), and 1,000 hPa (*n* = 13). Because images were acquired every 10 s, values near 5 s represent the practical lower temporal limit of this analysis. Data were collected from cells isolated from at least three animals per condition. The values adjacent to the bars represent the number of independently recorded single-cell observations. Statistical analysis was conducted using the Kruskal–Wallis test followed by Dunn’s *post hoc* test (Bi; **p < 0.005), Kruskal–Wallis followed by Tukey’s *post hoc* test (Bii; ***p < 0.001), and Kruskal–Wallis (C; *p* = 0.962).

We examined astrocyte [lactate]ᵢ dynamics after pressure-induced mechanical stimulation. Normalized FRET time courses (mean ± standard error of the mean [SEM]) for single stimuli are shown in Figure 6Ai (5 and 500 hPa) with a representative recording illustrating the initial lactate increase at 500 hPa. Normalized FRET time courses (mean ± SEM) for multiple sequential stimuli are shown in Figure 6Aii, with a representative recording at 500 hPa highlighting both the initial increase and subsequent decay phase (fall time).

Maximal rates of initial maximal [lactate]ᵢ increase (Figure 6B) were determined from the steepest 20-s window within 200 s post stimulus. For single stimuli (Figure 6Ai), rates were 0.57% ± 0.12%/min (*n* = 7), 0.64% ± 0.17%/min (*n* = 6), 1.41% ± 0.18%/min (*n* = 7), and 2.18% ± 0.65%/min (*n* = 8) at 5, 50, 500, and 1,000 hPa, respectively. Only rates at 5 versus 1,000 hPa differed significantly (Kruskal–Wallis: **p < 0.005, *H* = 13.04, df = 3; Dunn’s *post hoc* test: **p < 0.005, *Q*_(5 vs 1,000 hPa)_ = 2.98).

For sequential stimuli (Figure 6Aii), rates (calculated as the maximal increase within the first 10 s post stimulation) were 0.30 ± 0.18%/min, 2.51 ± 0.56%/min, 13.16 ± 1.42%/min, and 14.35 ± 2.09%/min (*n* = 15 each). These were consistently higher than single-stimulus rates due to transient spikes and differed significantly between 5 and 50 hPa compared to 500 hPa and 1,000 hPa (Kruskal–Wallis: ***p < 0.001, H = 40.32, df = 3; Tukey’s: q_(5 vs 1,000 hPa)_ = 7.38, q_(50 vs 1,000 hPa)_ = 5.25, q_(5 vs 500 hPa)_ = 7.08, q_(50 vs 500 hPa)_ = 4.95).

For sequential stimulation, transient lactate spikes were followed by rapid relaxation toward a new elevated stationary level. We quantified this relaxation as an apparent fall half-time, defined as the time required for the signal to move halfway from the transient spike maximum toward the subsequent stationary level. Because images were acquired every 10 s, apparent fall half-times close to 5 s are at the practical lower temporal limit of the analysis and should not be interpreted as precisely resolved sub-10-s kinetic constants. The apparent fall half-times were 5.56 ± 0.56 s at 50 hPa (*n* = 9), 6.54 ± 1.54 s at 500 hPa (*n* = 13), and 5.39 ± 0.39 s at 1000 hPa (*n* = 13), with no significant difference among pressures (Kruskal–Wallis: *p* = 0.962, H = 0.08, df = 2; Figure 6C). These values indicate that the transient component relaxed rapidly, within approximately one acquisition interval, to a sustained elevated plateau.

### 3.6 TRPV4 channel inhibition attenuates [lactate]_i_ responses to mechanical stimulation by a bolus application

**Figure 7:**
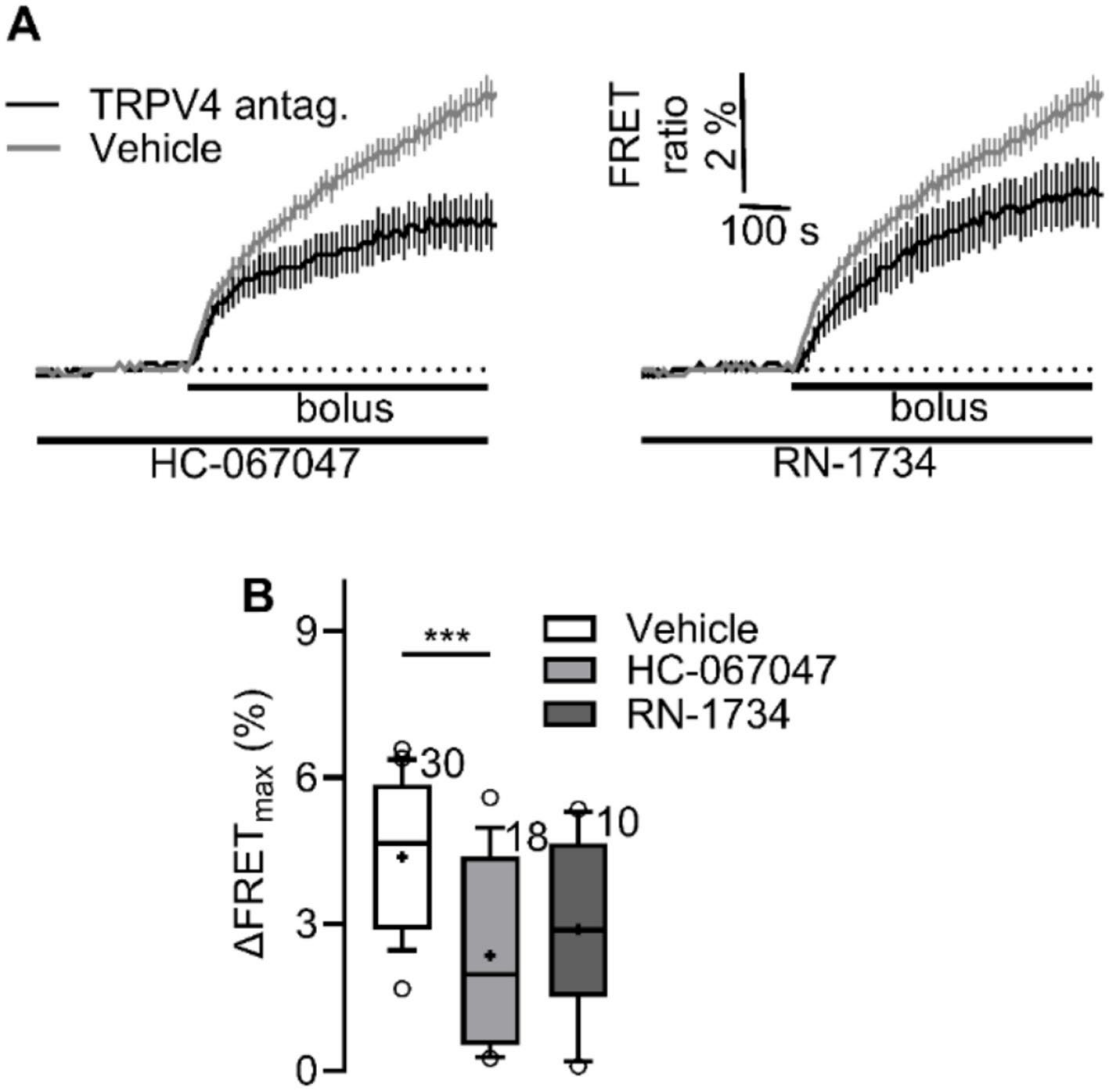
Pharmacologic inhibition of TRPV4 mechanosensitive channels reduces the increase in [lactate]_i_ after mechanical stimulation by a bolus application. **(A)** Mean ± SEM normalized FRET ratio time courses in astrocytes preincubated for 30 min with either TRPV4 channel antagonists (HC-067047, left panel; RN-1734, right panel) or vehicle (extracellular solution). Mechanical stimulation was applied via the addition of a manual bolus of the same solution used for preincubation. **(B)** Median maximal [lactate]_i_ amplitudes after stimulation with vehicle (*n* = 30), HC-067047 (light gray, *n* = 18), or RN-1734 (dark gray, *n* = 10). Data represent independent observations of individually recorded cells from at least three different animals. Statistical significance was assessed using the Kruskal–Wallis test (***p < 0.001), followed by Dunn’s *post hoc* test.

We applied two antagonists, HC-067047 (Figure 7A, left) and RN-1734 (Figure 7A, right), to assess TRPV4 channel involvement in the mechanically induced increase in [lactate]ᵢ. The figures show the mean ± SEM normalized FRET ratio time courses after the addition of a manual bolus of preincubation solutions under constant temperature, ensuring that responses reflect activation of mechanosensitive channels. These experiments were designed to test whether a mechanically induced lactate response is sensitive to TRPV4 inhibition. They do not directly test the TRPV4’s contribution to the sequential pressure-pulse protocol shown in Figure 4.

Astrocytes were preincubated for 30 min either in vehicle (extracellular solution) or antagonist solutions (10 μM HC-067047 or 10 μM RN-1734). After an initial 300-s baseline recording period, cells were stimulated using the solution in which they had been incubated, followed by 600 s of FRET recording. Sensor functionality was confirmed at the end of each recording by adding 20 mM lactate solution and observing responses for an additional 300 s (data not shown).

This experimental setup with bolus application elicited prominent mechanical-like responses in vehicle-treated cells, resulting in a 4.36% ± 0.28% increase in the FRET ratio (*n* = 30) at *t* = 600 s in the post-stimulus recording. Conversely, cells pretreated with HC-067047 or RN-1734 exhibited reduced increases in [lactate]_i_ of 2.36% ± 0.43% (*n* = 18) and 2.89% ± 0.53% (*n* = 10), respectively. Although both antagonists reduced [lactate]ᵢ production, only the reduction by HC-067047 reached statistical significance (Figure 7B; Kruskal–Wallis test; ***p < 0.001, *H* = 14.09, df = 2, followed by Dunn’s *post hoc* test; *p* < 0.05, *Q*_veh. vs HC-067047_ = 3.55).

## 4 Discussion

This study demonstrates that mechanical forces acting on astrocytes are rapidly converted into a surge of [lactate]_i_, partly through TRPV4-associated mechanosensitive signaling, thereby linking mechanotransduction to astrocyte metabolic support. It is known that astrocytes are highly responsive to mechanical stimulation, which activates complex signaling cascades involving mechanosensitive TRPV4 channels and connexin hemichannels (Turovsky et al., 2020). Increased vascular pressure, reflecting higher resistance to blood flow, typically elicits smooth muscle contraction, a response known as the myogenic mechanism (Golding et al., 1998). In contrast, the metabolic mechanism involves vasodilation under hypoxic conditions and vasoconstriction in the presence of oxygen (Johnson, 1986). Astrocyte endfeet are positioned at the blood vessels. This makes them unique in detecting mechanical stress, such as shear forces resulting from vascular constriction or dilation (Dunn et al., 2013). Activation of mechanosensitive TRPV4 channels, highly expressed at the astrocyte endfeet near blood vessels and brain surfaces (Benfenati et al., 2007; Dunn et al., 2013; Filosa and Iddings, 2013; Haidey et al., 2021), leads to increases in intracellular Ca^2+^ (Kim et al., 2015). TRPV4-mediated astrocytic Ca^2+^ signaling has been implicated in vascular regulation, but its physiological interpretation depends strongly on vascular context, stimulation modality, and timescale. Haidey et al. (2021) showed that increased arteriole tone can recruit TRPV4-mediated Ca^2+^ elevations in astrocyte endfeet and engage COX-1-dependent feedback regulation of ultra-slow arteriole oscillations. This study supports the concept that perivascular astrocytes can detect vascular mechanical state through TRPV4-associated signaling. However, ultra-slow spontaneous vasomotion is not equivalent to fast activity-induced functional hyperemia, and chronic genetic manipulation of astrocytic Ca^2+^ signaling may be influenced by compensatory mechanisms. Conversely, recent work by Vittani et al. (2025) showed that optogenetically induced elevation of astrocytic cAMP can dilate cerebral arterioles independently of IP3R2-mediated Ca^2+^ signaling (Vittani et al., 2025). Together, these studies indicate that astrocyte-dependent vascular regulation is not mediated by a single universal pathway, but depends on messenger identity, vascular segment, brain state, stimulation modality, and timescale. In the present study, we did not measure vascular diameter or cerebral blood flow; rather, we show that mechanical stimulation evokes intracellular lactate accumulation in astrocytes and that this response is attenuated by TRPV4 inhibition. Thus, our data support a TRPV4-sensitive astrocytic mechano-metabolic response while remaining compatible with the broader view that astrocytic vascular and metabolic signaling may involve both Ca^2+^-dependent and Ca^2+^-independent pathways. Fluctuations in systemic blood pressure, intracranial pressure, ischemia, trauma, or vascular tone can alter perfusion and mechanical stress in the brain (Patel et al., 2023). The astrocytic response to these stimuli is vital for maintaining homeostasis and protecting against pathologic states, underscoring their central role in cerebral blood flow regulation (Marina et al., 2020). Although not addressed experimentally in this paper, it is likely that fluctuations in intracranial pressure during cerebral perfusion, which stress perivascular astrocytes, may increase aerobic glycolysis and L-lactate production, thereby increasing astrocyte [lactate]_i_.

The glymphatic system hypothesis, although still debated (Jin et al., 2016; Smith et al., 2017; Thomas, 2019), posits that astrocytes shrink during sleep, thereby expanding perivascular spaces and facilitating cerebrospinal fluid flow. The increased flow, driven by blood pressure pulses, removes waste products from the brain (Jessen et al., 2015). The present data support the concept that astrocytes can convert mechanical cues into sustained intracellular metabolic responses. This interpretation is physiologically relevant because astrocytes occupy a central position in the neurovascular unit, where they are exposed to changes in vascular diameter, pressure, flow, and perivascular fluid movement (Iadecola and Nedergaard, 2007; Attwell et al., 2010; Marina et al., 2020). Rather than viewing the response as simply “advantageous,” we propose that mechanically triggered lactate accumulation may represent a cell-autonomous component of astrocytic adaptation to vascular or perivascular mechanical cues (Kim et al., 2015; Turovsky et al., 2020; Haidey et al., 2021). This concept is consistent with previous evidence that astrocytes sense cerebral perfusion, regulate vascular function, and modulate aerobic glycolysis and lactate production through intracellular signaling pathways (Pellerin and Magistretti, 1994; Marina et al., 2020; Fink et al., 2021; Horvat et al., 2021; Zorec and Vardjan, 2023; Fink et al., 2025).

Understanding this mechanotransduction system holds significant therapeutic implications. The glymphatic perivascular circulation, driven by astrocytic AQP4, effectively drains solutes from the brain, and dysfunctions in this pathway may critically contribute to neurodegenerative diseases (Li et al., 2024). The results of this study demonstrate that astrocytes function as active, mechanosensitive cells that regulate metabolism through L-lactate production and may contribute to the glymphatic system.

Astrocyte volume regulation may also be relevant to the present findings. Mechanical stimulation, TRPV4 activation, AQP4-dependent water flux, lactate/H^+^ transport, and intracellular osmotic balance are functionally interconnected in astrocytes. The AQP4/TRPV4 complex has been implicated in regulatory volume decrease after osmotic swelling (Benfenati et al., 2011), and lactate transport through monocarboxylate transporters is coupled to H^+^ movement and depends on transmembrane concentration and pH gradients (Hertz and Dienel, 2005). Therefore, mechanically induced lactate accumulation could, in principle, be associated with changes in astrocyte volume. Conversely, mechanical or osmotic changes in astrocyte volume could influence lactate production, intracellular dilution, MCT-dependent export, or putative channel-mediated lactate release. The present experiments were not designed to quantify cell volume: we did not acquire calibrated three-dimensional morphology, membrane labeling, or z-stack measurements. We therefore cannot determine whether the observed intracellular increase in lactate was accompanied by swelling or regulatory volume responses.

We exposed astrocytes to patch-micropipette–controlled pressure pulses and measured [lactate]_i_, an energy substrate and a signal (Pellerin and Magistretti, 1994; Gordon et al., 2008; Karagiannis et al., 2016) in real time by fluorescence microscopy and L-lactate nanosensors. Our findings demonstrate that a 1.5-s mechanical pressure pulse induces a sustained, long-lasting increase in [lactate]_i_. This response was dose-dependent; higher pressures elicited greater increases in [lactate]_i_. The absence of a return to baseline during the recording period is an important feature of the response. Laconic reports intracellular astrocytic lactate and does not distinguish between increased lactate production, reduced lactate clearance, altered transporter-mediated exchange, or changes in intracellular lactate buffering. Astrocytic lactate dynamics are governed by the balance among glycolytic production, glycogen-derived substrate flux, mitochondrial metabolism, MCT-dependent transport, and possibly channel- or hemichannel-mediated lactate release (Sotelo-Hitschfeld et al., 2015; Fink et al., 2021; Horvat et al., 2021). In an intact neurovascular unit, the extracellular lactate profile would likely be reshaped by MCT-dependent astrocyte-neuron exchange and vascular removal (San Martín et al., 2013).

When applying short pressure pulses in an increasing sequence, distinct transient increases (spikes) in [lactate]_i_ were recorded, suggesting cumulative effects or sensitization. New stationary states followed these spikes, and single stimulations led to more gradual increases in [lactate]_i_. Although the nature of the spikes is unclear, they may likely be due to glycogen-derived transient degradation followed by transporter-limited lactate clearance (Hertz et al., 2014). Recent *in vivo* work by Stelzner et al. (2024) provides a useful biological context for this distinction. Using two-photon imaging in awake mice, they related astrocytic Ca^2+^ activity and vascular responses to volitional whisking, experimenter-evoked whisker stimulation, and whisking preceding locomotion. In this framework, our brief 1.5-s pressure pulse can be viewed as a simplified *in vitro* analogue of an acute mechanical component accompanying short sensory-evoked vascular responses, whereas the sequential protocol may better approximate repeated or prolonged hemodynamic engagement during behavioral states.

The functional interpretation of the mechanically induced rise in intracellular lactate requires distinction between astrocytic lactate production and astrocyte-to-neuron lactate transfer. In the present study, Laconic reports intracellular lactate in isolated primary astrocytes; therefore, our data does not directly demonstrate extracellular lactate release, neuronal uptake, or activation of the astrocyte-neuron lactate shuttle. Rather, the results identify a cell-autonomous astrocytic mechanism by which brief mechanical stimulation increases the cytosolic lactate pool. In intact brain tissue, such an increase could contribute to lactate availability for neurons if coupled to export through monocarboxylate transporters or channel-mediated pathways. This interpretation is consistent with *in vivo* evidence for a lactate gradient from astrocytes to neurons and with the concept that lactate acts not only as a fuel but also as a signaling molecule regulating neuronal excitability, plasticity, and memory-related processes (Mächler et al., 2016; Magistretti and Allaman, 2018). Thus, mechanically triggered intracellular lactate accumulation may represent an upstream astrocytic component of metabolic support, although direct lactate shuttle activity remains to be tested in neuron-containing preparations.

The present study also does not determine whether mechanically induced astrocytic lactate accumulation alters neuronal excitability. Our purified astrocyte culture model was selected to isolate the astrocytic response to mechanical stimulation without confounding contributions from neuronal firing, synaptic transmission, vascular cells, or neuronal lactate consumption.

Mechanical sensitivity analysis revealed an estimated activation threshold for the increase in [lactate]_i_ of approximately 3–5 hPa across stimulation protocols. This low threshold is the most physiologically relevant aspect of the pressure-response analysis because it lies within the range of small pressure fluctuations that may occur in mechanically active perivascular environments. In contrast, the 500 and 1,000 hPa command pressures should be considered supraphysiological mechanical challenges. They were included to establish the pressure-response relationship and response kinetics over a broad dynamic range. The brain, although enclosed within the rigid skull, remains vulnerable to pressure fluctuations and shear forces from changes in blood flow, trauma, or ischemia (Rosenegger and Gordon, 2015). Low-amplitude intracranial-pressure oscillations (spindle waves) may reach roughly 5 hPa (≈4 mmHg) (Zhu et al., 2022), and these small pressure changes may activate glycogen stores in astrocytes to rapidly generate increases in lactate, as supported by our previous findings linking lactate production to glycogenolysis (Fink et al., 2021; Fink et al., 2025).

Our results also highlight the relatively rapid kinetics of the lactate response, with stimulus intensity correlating with the rate of increase in [lactate]_i_. Sequential pressure pulses yielded faster rates than single pulses, likely due to the presence of initial spikes and/or signal conditioning, which needs to be studied further. These responses typically reached a new steady-state with a fall time of approximately 10 s, reflecting compensatory normalization mechanisms.

Previous studies have reported TRPV4 expression in astrocytes, but its distribution appears heterogeneous. TRPV4 channels are present in cortical astrocytes (Benfenati et al., 2007; Kim et al., 2015), but findings in hippocampal astrocytes are inconsistent (Shibasaki et al., 2014; Pivonkova et al., 2018; Toft-Bertelsen et al., 2018; Diaz et al., 2019). In some cases, TRPV4 expression has been confirmed only under pathologic conditions such as hypertension or ischemia (Butenko et al., 2012; Diaz et al., 2019), often associated with astrogliosis. In this study, we confirmed the presence of TRPV4 in cultured primary rat cortical astrocytes. Moreover, pharmacologic inhibition of TRPV4 with HC-067047 significantly attenuated [lactate]_i_ responses (by ~45%), whereas RN-1734 showed a similar, but statistically non-significant reduction (~34%). The effect of TRPV4 inhibition was partial. HC-067047 significantly attenuated the mechanically induced lactate response, whereas RN-1734 produced a similar but statistically non-significant reduction. These data support TRPV4 contribution but do not establish TRPV4 as the sole or dominant mechanotransduction pathway. Astrocytes express several mechanosensitive or mechanically recruited signaling systems, including PIEZO1 channels (Velasco-Estevez et al., 2020; Chi et al., 2022), pannexin channels (Beckel et al., 2014), connexin 43 hemichannels, purinergic P2Y signaling, and AQP4/TRPV4-associated volume-regulatory complexes (Benfenati et al., 2011). These pathways may interact; for example, mechanical stimulation can recruit TRPV4/Cx43-dependent ATP release and downstream purinergic amplification of astrocytic Ca^2+^ signaling (Turovsky et al., 2020). Therefore, the mechanically induced lactate response observed here is best interpreted as TRPV4-sensitive but not exclusively TRPV4-mediated. Because TRPV4 antagonists were tested using bolus mechanical stimulation rather than the sequential micropipette pressure-pulse protocol, the pharmacological results should be interpreted as evidence that TRPV4 contributes to mechanically induced lactate accumulation, but not as proof that TRPV4 accounts for all components of the sequential-pulse response.

Direct activation of TRPV4 with an agonist such as GSK1016790A may be useful for testing whether pharmacological TRPV4 opening is sufficient to alter astrocytic lactate metabolism (Harraz et al., 2018). However, agonist application would not reproduce the localized and transient nature of the pressure-pulse stimulus. In addition, GSK1016790A evoked robust Ca^2+^ influx, which could confound lactate measurements by inducing Ca^2+^ overload.

In the present study we did not directly image intracellular Ca^2+^ or Na^+^ during pressure-pulse stimulation. Astrocytic metabolic regulation is likely not a simple linear pathway, because it may involve Ca^2+^, Na^+^-dependent transport, cAMP signaling, glycogen remodeling, monocarboxylate transporters, and channel- or hemichannel-mediated lactate flux. Moreover, Ca^2+^ and cAMP signaling can interact through Ca^2+^/calmodulin-sensitive adenylyl cyclases, as shown for AC1 and AC8 by Masada et al. (2009). Future studies combining Ca^2+^, cAMP, and lactate imaging under identical mechanical stimulation protocols will be required to define the temporal order of these events.

Our findings also emphasize methodological consideration; manual addition of solution can itself induce substantial mechanical stimulation, producing [lactate]_i_ responses comparable with those elicited by 1,000 hPa pressure pulses. This potential confounding issue underscores the importance of careful control conditions in studies evaluating pharmacologic modulation of astrocyte metabolism.

## 5 Conclusion

Our results show that astrocytes exhibit a robust metabolic response to mechanical stimulation, marked by a rapid, sustained increase in [lactate]_i_. This response occurs in relation to the stimulus intensity and exhibits distinct kinetics depending on whether stimulation is delivered as a single pulse or sequential pressure pulses. The results further demonstrate that TRPV4 channels, at least in part, mediate this mechanosensitive lactate production because pharmacologic inhibition with HC-067047 significantly attenuated the increase in [lactate]_i_. These findings support a model in which astrocytes function as mechano-metabolic sensors, linking mechanical cues to intracellular lactate accumulation. In the intact neurovascular unit, this astrocytic response may increase the lactate pool available for export and neuronal use, although direct astrocyte-to-neuron lactate transfer was not tested in the present monoculture system.

## Data Availability

The raw data supporting the conclusions of this article will be made available by the authors, without undue reservation.
