# Supplementary material for: Brief mechanical pulses induce sustained intracellular L-lactate production in astrocytes
Source: Front Cell Neurosci. 2026 Jun 17;20:1830831. doi: 10.3389/fncel.2026.1830831 (PMC13319024; doi:10.3389/fncel.2026.1830831)
Supplement: Supplementary file 1 [file Supplementary_file_1.pdf]

Supplementary Material

Brief mechanical pulses induce sustained intracellular L-lactate production in astrocytes

Kaja Belko Parkel, Danaja Kuhanec, Živa Tajda Gržina, Helena Haque Chowdhury, Robert Zorec, and Marko Kreft

Supplementary Figures

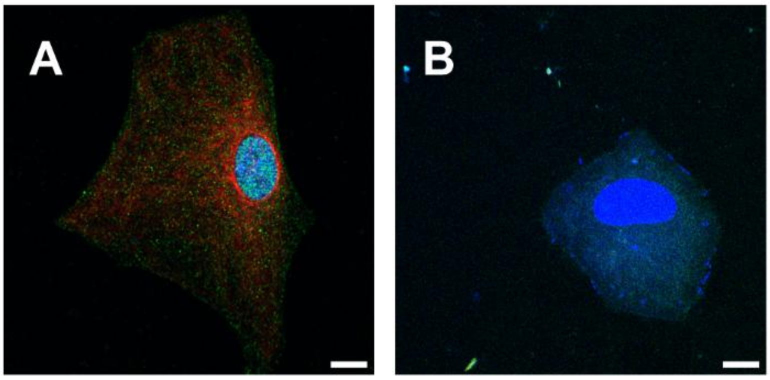

Supplementary Figure S1. Immunofluorescence detection of TRPV4 and GFAP in primary rat astrocytes.
(A) Representative immunofluorescence image of a primary rat astrocyte showing TRPV4 staining in green and glial fibrillary acidic protein (GFAP), an astrocytic marker, in red. Cell nuclei were counterstained with DAPI and are shown in blue.
(B) Negative control processed in parallel without primary antibodies, showing minimal non-specific secondary-antibody fluorescence. DAPI nuclear staining is shown in blue. Scale bars: 10 µm.

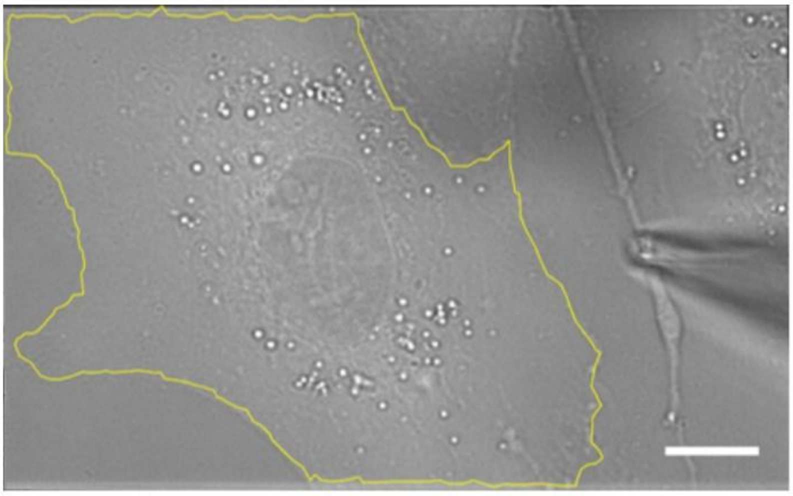

Supplementary Figure S2. A glass micropipette placed above an astrocyte was used for the mechanical stimulation
Representative transmitted light image showing a primary rat astrocyte outlined by a yellow line indicating the cell perimeter. A glass micropipette positioned on the right hand side is used for controlled mechanical stimulation. Scale bar: 10 µm.
